# Total joint replacement of the hip and knee in patients with arthrogryposis multiplex congenita: a report of six joints

**DOI:** 10.1007/s00402-020-03611-3

**Published:** 2020-10-10

**Authors:** Christoph Theil, Robert Roedl, Georg Gosheger, Burkhard Moellenbeck, Adrien Frommer, Ralf Dieckmann, Bjoern Vogt

**Affiliations:** 1 Department of Orthopaedics and Tumour Orthopaedics, Muenster University Hospital, Muenster, Albert-Schweitzer-Campus 1, 48149 Münster, Germany; 2 Divison of Children’s Orthopaedics, Deformity Correction and Foot Surgery, Muenster University Hospital, Muenster, Albert-Schweitzer-Campus 1, 48149 Münster, Germany

**Keywords:** Arthrogryposis, Arthroplasty, THA, TKA

## Abstract

**Introduction:**

Arthrogryposis multiplex congenita (AMC) is a rare congenital condition that leads to severe joint contractures and deformities. As painful joint dysplasia and degeneration might develop over time, total joint replacement (TJR) can be a potential treatment option for these patients. The aim of this study is to investigate functional results, implant survivorship and potential complications in patients with AMC who undergo hip or knee arthroplasty.

**Materials and methods:**

We retrospectively identified six TJR in three patients at a single centre performed between 2006 and 2019. The median patient age at surgery was 23 years and the median follow-up period was 69 (IQR 55–99) months. We analysed surgical technique, implant survivorship and complications as well as functional outcome determined by pain reported on the Numerical Rating Scale (NRS), patient-reported outcome scores [Oxford Hip Score (OHS), Harris Hip score (HHS), Oxford Knee Score (OKS)], range of motion and ambulatory status. Depending on data distribution means with ranges and median with interquartile range were compared with the Wilcoxon signed rank test or Student’s *t* test. The level of significance was defined at < 0.05.

**Results:**

In hips, the mean range of motion in flexion/extension (52° vs. 85°, *p* = 0.014) and in rotation (28° vs. 68°, *p* = 0.02) as well as mean pain score on the NRS (8.5 vs. 0, *p* = 0.001), OHS (9 vs. 26, *p* = 0.031) and HHS (17 vs. 52, *p* = 0.007) significantly improved. In knees, mean range of motion (55° vs. 93°, *p* = 0.403), mean pain score on the NRS (0 vs. 7) and the OKS (2 vs. 21) also improved. While the ambulatory status did not change, the patients who were wheelchair dependent reported less problems with transfers to a bed or chair and the patient who ambulated reported an improved walking distance. One total knee arthroplasty (TKA) underwent revision for an acute, late infection 155 months following the initial surgery.

**Conclusions:**

TJR is a safe procedure in patients with AMC that effectively improves function and reduces pain irrespective of preoperative ambulatory status.

## Introduction

Arthrogryposis multiplex congenita (AMC) is a rare congenital symptom complex that describes a collection of aetiologies with several significant joint contractures and consequent deformities [1–3]. While the condition is uncommon, a high percentage of patients have relevant pathologies of the lower extremities, particularly of the hip and knee joints [2, 3]. Many arthrogryposis hips are dislocated or demonstrate severe flexion contractures which require surgical reduction or release during childhood and adolescence [1, 2, 4, 5]. In more severe cases or recurrent dislocation, corrective osteotomies may be required [4]. The knee joints commonly display flexion or extension contractures which can be corrected by soft tissue release or extension osteotomies during childhood and adolescence [1, 2].

Considering that a high percentage of patients with AMC has the potential to ambulate in adulthood and has a close to normal life expectancy [6, 7], surgical intervention to correct deformities or to release contractures should be performed to maintain or regain long-term ability to ambulate and to prevent or relieve pain [2, 7]. However, due to the often severe and recurring contractures and deformities [1, 5, 8] some patients may develop degenerative joint changes that cause pain and limited function [9].

Considering the perpetual improvements in total hip arthroplasty (THA) and total knee arthroplasty (TKA) that allow for simultaneous correction of deformities and show excellent long-term survival [10–12], these surgeries may be considered as a potential treatment option in patients with AMC who suffer from severe deformities and osteoarthritis changes.

However, THA and TKA following deformity correction in patients with syndrome associated joint dysplasia are usually technically challenging procedures and only few solitary cases have been reported so far [9, 13, 14].

The aim of the present study is to analyse the surgical treatment details, implant survivorship and functional results following THA or TKA in patients suffering from AMC and provide an overview of the current literature.

## Methods

We conducted a retrospective database search of a single institution’s electronic database and identified a total of 18 consecutive patients who underwent surgical treatment at our department for AMC between 2006 and 2019.

Of these patients, three received total joint replacement (TJR). All of whom had tetramelic amyoplasia type 1 according to the Munich classification (disorders with mainly limb involvement) [15]. Two patients underwent unilateral THA while one patient received staged bilateral TKA and bilateral THA. Therefore, six TJRs at a median patient age of 23 years (IQR 17.5–24.3) in three patients could be reviewed and were included in this study.

TJR surgery was indicated if pain and joint degeneration was present and conservative treatment with non-steroidal anti-inflammatory drugs and physical therapy had failed. All patients previously underwent multiple surgical interventions with soft tissue releases to address contractures and correction of clubfoot deformities in our department and in other hospitals. However, in only one patient to our knowledge a subtrochanteric varisation osteotomy and modified acetabuloplasty were performed [16] to treat a hip dislocation during childhood.

**Fig. 1:**
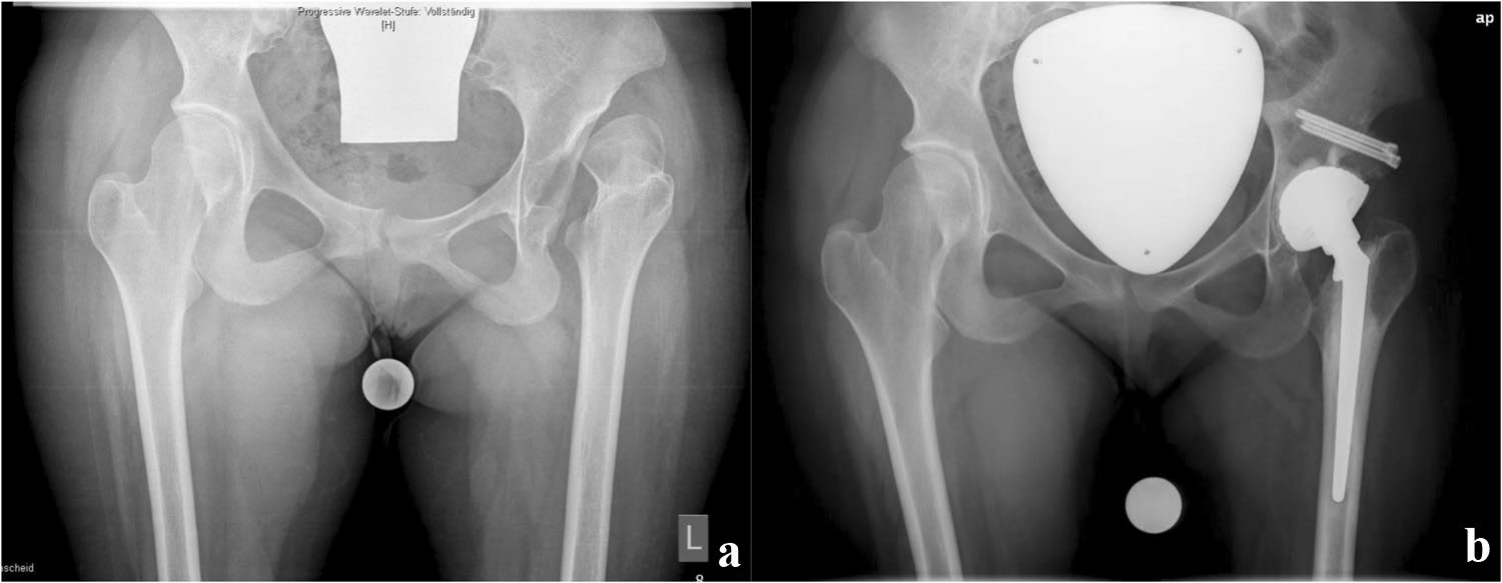
**a** Preoperative radiograph showing chronic hip dislocation on the left with osteoarthritic changes. **b** Postoperative radiograph after THA and concomitant acetabular augmentation with the femoral head

The THA (exemplary Fig. 1a, b) were executed through a lateral approach. In one patient with a painful chronic dislocation a cemented dysplastic hip stem (CDH stem, Implantcast GmbH, Buxtehude, Germany) with a dual-mobility cemented acetabular cup (Ecofit 2M, Implantcast GmbH, Buxtehude, Germany) was used and was combined with acetabular augmentation using the femoral head [17]. The other three cases were not dislocated and were addressed using a small cementless stem with diaphyseal anchorage (Dialoc, Implantcast GmbH, Buxtehude, Germany) and a cementless porous coated shell with additional screw fixation on the acetabular side (Trident, Stryker Corp., Kalamazoo, MI, USA). All patients underwent extensive arthrolysis and tenotomies to facilitate the joint replacement surgery and to treat contractures.

**Fig. 2:**
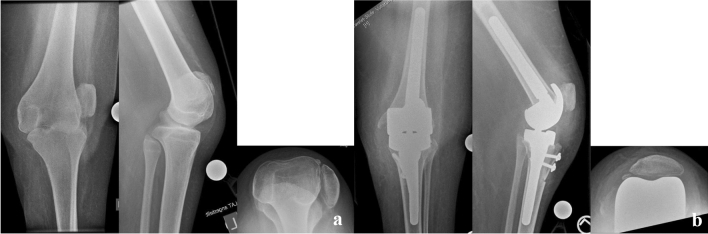
**a** Preoperative radiograph of the painful knee in subluxation. **b** Postoperative radiograph following rotating hinge knee replacement

For TKA (exemplary Fig. 2a, b), a medial extensile approach was chosen and concomitant tendon releases were executed. One TKA was performed with a stemmed condylar constrained implant system (Genesis CC, Smith&Nephew Inc., Memphis, TN, USA) and the other with a stemmed rotating hinge knee system due to severe laxity and dysplasia of the femoral condyles (GenuX, Implantcast GmbH, Buxtehude, Germany) which was combined with a tibial tuberosity proximalisation.

We recorded patient details, previous surgeries, surgical details, implant survivorship and functional results from the patient’s files. Implant survivorship was calculated to the last follow-up date or revision surgery. Periprosthetic joint infection (PJI) was diagnosed using the criteria as proposed by the musculoskeletal infection society (MSIS) published in 2018 [18]. Functional results were assessed using the preoperative and postoperative Harris Hip Score (HHS) [19] and Oxford Hip Score (OHS) [20] for THA and the Oxford Knee Score (OKS) [21] for TKA. The worst possible score in the Oxford scores is 0 and the best possible score is 48. For the HHS the worst possible score is 0 and the best possible score is 99. All of these scores are patient-reported outcome scores (PROMS), only the HHS requires physician assessment of gait and range of motion. Furthermore, pre- and postoperative range of motion was measured, pain level was determined on the Numeric Rating Scale (NRS) from 0 to 10, general ability to walk and the use of a wheelchair or other walking aids were recorded. All results were collected at the latest follow-up appointment.

### Statistical analysis

We conducted a descriptive statistical analysis and analysed the distribution of data using the Shapiro–Wilk test. For non-parametric data median and 25–75% interquartile ranges (IQR) were presented while for parametric data means and ranges were given. Differences in functional scores were compared using the Wilcoxon signed rank test for non-parametric data and the Student’s *t* test for paired, parametric data. Level of significance was defined at < 0.05; all *p* values were two sided.

## Results

The median follow-up period amounted to 69 months (IQR 53–99) with all patients available for functional analysis.

In the four THA performed pain, range of motion and functional scores all improved significantly. The preoperative hip flexion and extension range was at a mean of 52° (range 40–70) preoperatively and 85° (range 70–90) postoperatively (*p* = 0.014) and the range of motion in internal and external hip rotation improved from a mean of 28° (range 10–50) to 63° (range 40–80) postoperatively (*p* = 0.002).

The mean pain score as determined by the NRS fell to a mean value of 0 postoperatively from a preoperative mean pain score of 8.5 (range 7–10) (*p* = 0.001).

The preoperative OHS improved from a mean of 9 (range 3–13) to 26 (range 20–40) postoperatively (*p* = 0.031) and the HHS from a mean 17 (range 0–32) to 52 (range 40–74) postoperatively (*p* = 0.007).

The two TKA resulted in an improvement of knee flexion and extension from 55° (range 40–70) preoperatively to 93° (range 80–105) postoperatively (*p* = 0.403). The NRS fell from 7 to 0 and the OKS improved from a mean of 2 (range 1–2) to 21 (range 20–21). Testing of the significance was not possible because the difference in standard deviation was 0.

Two patients who were wheelchair users remained wheelchair dependent postoperatively due to muscle contractures. However, the ability to transfer between chairs or to a bed improved postoperatively. One patient who was ambulatory prior to THA remained ambulatory with greatly improved walking distance.

While all TJR were initially successful with no early complications (fracture, neurovascular injuries) or mechanical complications such as dislocation or loosening, one patient developed a late acute PJI of the replaced knee joint 155 months following primary arthroplasty. Symptoms with newly onset knee pain and swelling occurred several weeks after a foot pressure ulcer due to an increasing ankle contracture on the same leg. Cultures yielded *Streptococcus pyogenes* in all samples. The patient initially underwent debridement, irrigation and exchange of the mobile inlay. However, 6 weeks after the initial revision a fistula occurred and explanation of the prosthesis with placement of an antibiotic loaded spacer was performed. The cultures at the first-stage of the planned two-stage exchange yielded methicillin-resistant* Staphylococcus epidermidis* and the patient currently undergoes antibiotic treatment.

### Case description 1

Patient 1 initially presented to our arthroplasty department at the age of 16 due pain and 30° of flexion contracture of the right knee. Prior to this, at age 9 he had undergone open arthrolysis that was initially successful but had recurred over the last 2 years prior and was associated with disabling pain despite the limited overall mobility with dependency on a wheelchair from early on. Additionally the patient had undergone complex corrective hind foot surgery to correct bilateral clubfoot deformities in early childhood and requires orthopaedic shoes since then. The patient was consulted for either re-arthrolysis or arthrolysis and joint replacement because of the fact that the primary issue was pain at mobilisation and transfers. The postoperative rehabilitation resulted in a range of motion at discharge of full extension with 50° of active flexion. Wheelchair transfers were consequently possible. At long-term follow-up, the range of motion improved to 110° of flexion. Unfortunately, the patient underwent treatment for PJI of this TKA later on as described above.

After 5 years of uneventful follow-up visits at an outside orthopaedic practice, the patient presented at age 21 with a laterally dislocated patella and subluxated, stiff knee in 30° of flexion contracture on the left side. The patient complained of worsening pain at weight bearing and during transfers. Due to the arthritic changes and successful surgery on the right side, a primary rotating hinge knee was implanted resulting in a postoperative range of motion of 90° flexion with 10° of residual flexion contracture. The patient was able to perform transfers and short periods of standing 3 months after surgery.

The patient presented again at age 23 with bilateral hip pain and was found to have dysplastic and arthritic hips. Once again, transfers were not possible independently and pain at rest was also reported. The patient had bilateral flexion contractures of 20° with maximum flexion of 60° and no rotatory motion due to pain. The left hip was replaced first using a cementless diaphyseal anchorage stem and a cementless primary hip cup. Due to the successful treatment with postoperative absence of pain and recovered motion and ability to do transfers over the left leg, the patient underwent replacement of the right hip using the same implants 8 months after the left side. After 6 weeks of non-weight bearing, the patient went on to full weight bearing and achieved improved flexion in both hips (Table 1).

**Table 1:** Patient details and range of motion at final follow-up

Patient and joint	Preoperative extension flexion	Postoperative extension flexion	Implants used	Approach
1, TKA left	0-30-70	0-5-110	Rotating hinge TKA	Medial parapatellar
1, TKA right	0-30-100	0-10-90	Condylar constrained TKA	Medial parapatellar
1, THA left	0-20-60	0-10-80	Diaphyseal stem, cementless cup	Lateral
1, THA right	0-20-60	0-0-90	Diaphyseal stem, cementless cup	Lateral
2 THA left	0-10-70	0-0-90	Diaphyseal stem, cementless cup	Lateral
3 THA left	0-0-70 (dislocated hip)	0-0-90	Dysplasia stem, cemented, cemented dual-mobility cup	Lateral

### Case description 2

Patient 2 first presented to our arthroplasty department at age 27 due to progressive left hip pain, limited range of motion of 70° degrees of flexion with 10° flexion contractures with no pain-free rotatory motion and reduced walking distance of less than 100 m. She had previously undergone varisation of the proximal femur and acetabuloplasty on the same side at age 5 for teratogenic hip dislocation. Since then the hip has been reduced. She had previously undergone multiple tenotomies of the lower extremity flexors around both knees as well as triple arthrodeses of both feet for recurrent clubfoot deformity at age 12.

The patient underwent primary THA using a cementless stem and cup through a lateral approach.

Postoperatively the patient regained full extension with flexion to 90° and 20° of internal and external rotation without pain. Ambulation without aids is in the domestic environment, but she still requires a wheelchair for outside activities.

### Case description 3

The third patient presented at age 18 due to worsening hip pain on the left side with reduced range of motion of 70° of flexion with full extension and no rotatory motion. The patient had been ambulatory since childhood despite a teratogenically dislocated hip on the left. The maximum walking distance had been reduce to 500 m while it was almost unlimited before. The patient had previously undergone several soft tissue releases and arthrolysis of the knee and ankle using external fixation at an outside hospital during childhood. The hip had not been operated on previously.

Due to the small femur configuration and reduced bone quality due to the teratogenic dislocation, a cemented dysplasia stem and a cemented dual-mobility cup was used. Additionally, a Harris plasty of the acetabulum was performed using the patient’s femoral head.

After 6 weeks of partial weight bearing to allow the Harris plasty to achieve bony fixation, the patient assumed full weight bearing and returned to unassisted ambulation with no pain and unlimited walking distance.

As for rehabilitation, the patients usually remained in the hospital for 1 week and went on to undergo conventional orthopaedic rehabilitation treatment when full weight bearing had been achieved.

## Discussion

While AMC is a serious disease that leads to severe impairment of joint function and consequently may restrict the ability to ambulate, overall life expectancy is generally considered to be close to normal and the treatment goal is to enable patients to live an independent life [1, 6, 7, 22]. In view of the above, some patients who suffered hip dislocation or knee subluxation due to AMC might go on to develop painful degenerative joint changes and contractures. Therefore, these patients are candidates for TJR when conservative treatment does not lead to sufficient improvement of symptoms, particularly osteoarthritic pain. To our knowledge, arthroplasty in patients with AMC has only been described in case reports so far; therefore, this study presents the only series of patients with AMC who underwent TJR that systematically investigates functional results. We were able to show that TJR of painful osteoarthritic hips and knees is a successful and durable reconstruction method in this particular patient cohort. However, there are several aspects to be discussed to guide patient expectations and to be aware of potential complications.

While this is the only case series in the literature that systematically investigates patients with AMC and patients were operated on in a single centre, this study has several limitations. First, it is a retrospective study that relies on the completeness of patient records and is prone to selection bias as patients might have undergone further treatment at other hospitals. However, probably due to the rarity of the disease all patients have returned to regular follow-up visits and the reported results came from very recent visits to our outpatient clinic. Second, while this is the largest collection of cases in the literature, it is still based on the results of only six TJR surgeries. Therefore, the presented results might not be applicable to every patient with AMC seeking advice on TJR. Third, while only one patient has developed a major complication and underwent reoperations, the limited number of patients that are available from our report and the overall literature make it difficult to identify risk factors for complications or assess the overall risk for revision surgeries following TJR. Fourth, while we report PROMS using several established scores to measure functional results in these patients, particularly in the Oxford scores, several questions were not addressed or scored with 0 out of 4 possible points (lowest possible results) by two of the patients who are still largely dependent on a wheelchair to handle the activities of daily living. In consequence, the test values were lower than expected despite both patients being subjectively very satisfied with the results. This must be considered when measuring function in patients who are not fully ambulatory.

In four previous reports (Table 1) [9, 14, 23, 24], the authors successfully performed TJR surgery in patients who were ambulatory and were primarily treated for osteoarthritic joint pain. All patients returned to full ambulation and improvement in functional scores with good long-term implant survival was reported. On the other hand, Cameron [13] reported on two THA and one TKA in one patient in whom the limitation of joint range of motion was of primary concern and concluded that the benefit from these procedures was doubtful as all joints returned to their previous deformity with limited range of motion within 2 years. However, for painful joints, particularly if dislocated and osteoarthritic, THA or TKA might still be a good treatment option. Cameron later described four cases of THA in patients with AMC [25] that were treated with modular hip stems among a larger patient cohort that was treated with this specific stem design. Unfortunately, there are no separate results presented on these cases. However, based on the results from the present study we can still recommend TJR surgery in patients who are not able to ambulate, but depend on a wheelchair as pain significantly improved postoperatively. Nonetheless, patients must be counselled regarding the high possibility of recurring joint contractures and the fact that the ability to ambulate might improve with reduced pain, but can still be very limited because of the boundaries set by range of motion (Table 2).

**Table 2:** Literature overview on TJR in patients with AMC

References	*N* Patients/joints	Follow-up in months	Surgical details	Clinical outcome	Complications
Dalton et al. [14]	One patient, two THA	84	Cementless, modular stem, standard acetabulum	No pain, limited ROM	None
Fisher et al. [9]	One patient, two TKA, two THA	6–120	Condylar constrained TKA, cementless, modular stem, standard acetabulum	No pain, improved ROM	Acetabular cup revision for aseptic loosening after > 10 years
Leonard et al. [23]	One patient, one THA	60	Cementless, modular stem, standard acetabulum	No pain, ROM at final follow-up not reported	None
Cameron et al. [13]	One patient, two THA, one TKA	Min. 24	Cementless, modular stem, standard acetabulum, condylar constrained TKA	Pain was not the main issue, ROM returned to previous levels, loss of ambulation	Heterotopic ossification, no revision surgery

Considering the young age of the patients who underwent TJR surgery in the present study, long-term implant survivorship may become an issue. The Nordic arthroplasty registry recently reported a 10-year survival probability of 83% for THA in patients under 21 years of age with the most common indication being inflammatory arthritis and aseptic loosening being the main reason for revision [26]. This is comparable to a study by Busch et al. [27] who published the same probability at 10 years in patients younger than 30 years of age at the time of THA and Clohisy et al. [28] who reported a 9% complication rate at close to 6 year follow-up period in patients 25 years of age and younger. All authors conclude that both cemented and uncemented reconstructions are possible and long survival can be achieved. However, patients must counselled regarding the risk of future revisions. Considering that a certain percentage of patients with AMC does not ambulate, it remains unclear what the long-term survivorship of joint arthroplasties will be assuming a comparatively low wear of the implants. The only complication in our cohort was one case of PJI, possibly due to a haematogenous spread following an ipsilateral skin ulceration. This problem was also addressed by Leonard and Nicholson [23] who reported on a patient with AMC that underwent THA after ipsilateral below the knee amputation for chronic ulcerations. As many patients with AMC will continue to have contractures and residual joint deformities even with maximal surgical and non-surgical care, the risk of ulcerations might increase the risk for haematogenous PJI [29] when TJR is performed and patients should be counselled regarding the need for skin care and preventive actions.

Lastly, as dislocation in THA might be a particular risk in AMC patients due to the joint contractures and risk for recurrent contractures of the affected muscles after surgery, dual-mobility cups [30] may be used if intraoperative stability appears compromised or the hip was previously dislocated. However, this appears to be an individual decision with several successful THAs reported without the use of dual-mobility cups in our series as well as in previous studies [13, 14, 23, 25]. Additionally, subtrochanteric shortening osteotomies might help to reduce soft tissue tension although this was not necessary in the present series and can be associated with higher rate of complications [31, 32]. However, for dysplastic hips with high dislocation this might be a good option to enable hip reposition during arthroplasty.

To our knowledge, there are no comparable studies on TKA survival in very young patients; nonetheless, the same concerns might be transferred to TKA and instability could be addressed pre-emptively with the use of a constrained or hinged knee.

In conclusion, based on the results from the present study and the limited evidence provided in the literature, we recommend joint replacement surgery in patients with painful joint deformities and osteoarthritic changes due to AMC independent of the ambulatory status. Implant selection as well as fixation techniques can be adapted to the local joint configuration, bone quality and severity of soft tissue contractures. Long-term studies should address implant survivorship and complications in patients with AMC.

## Data Availability

The datasets used and/or analysed during the current study are available from the corresponding author upon reasonable request.
